# Genetic Diversity of Recent Infectious Bursal Disease Viruses Isolated From Vaccinated Poultry Flocks in Malaysia

**DOI:** 10.3389/fvets.2021.643976

**Published:** 2021-04-20

**Authors:** Hayatuddeen Bako Aliyu, Mohd Hair-Bejo, Abdul Rahman Omar, Aini Ideris

**Affiliations:** ^1^Department of Veterinary Clinical Studies, Faculty of Veterinary Medicine, Universiti Putra Malaysia, Serdang, Malaysia; ^2^Avian Unit, Veterinary Teaching Hospital, Ahmadu Bello University, Zaria, Nigeria; ^3^Laboratory of Vaccine and Biomolecules, Institute of Bioscience, Universiti Putra Malaysia, Serdang, Malaysia; ^4^Department of Veterinary Pathology and Microbiology, Faculty of Veterinary Medicine, Universiti Putra Malaysia, Serdang, Malaysia

**Keywords:** infectious bursal disease virus, variant IBDV, vvIBDV, chickens, next-generation sequencing

## Abstract

Vaccination is an essential component in controlling infectious bursal disease (IBD), however, there is a lack of information on the genetic characteristics of a recent infectious bursal disease virus (IBDV) that was isolated from IBD vaccinated commercial flocks in Malaysia. The present study investigated 11 IBDV isolates that were isolated from commercial poultry farms. The isolates were detected using reverse transcription-polymerase chain reaction (RT-PCR) targeting the hypervariable region (HVR) of VP2. Based on the HVR sequences, five isolates (IBS536/2017, IBS624/2017, UPM766/2018, UPM1056/2018, and UPM1432/2019) were selected for whole-genome sequencing using the MiSeq platform. The nucleotide and amino acid (aa) sequences were compared with the previously characterized IBDV strains. Deduced aa sequences of VP2HVR revealed seven isolates with 94–99% aa identity to very virulent strains (genogroup 3), two isolates with 97–100% aa identity to variant strains (genogroup 2), and two strains with 100% identity to the vaccine strain (genogroup 1) of IBDV. The phylogenetic analysis also showed that the isolates formed clusters with the respective genogroups. The characteristic motifs 222T, 249K, 286I, and 318D are typical of the variant strain and were observed for UPM1219/2019 and UPM1432/2019. In comparison, very virulent residues such as 222A, 249Q, 286T, and 318G were found for the vvIBDV, except for the UPM1056/2018 strain with a A222T substitution. In addition, the isolate has aa substitutions such as D213N, G254D, S315T, S317R, and A321E that are not commonly found in previously reported vvIBDV strains. Unlike the other vvIBDVs characterized in this study, UPM766/2018 lacks the MLSL aa residues in VP5. The aa tripeptides 145/146/147 (TDN) of VP1 were conserved for the vvIBDV, while a different motif, NED, was observed for the Malaysian variant strain. The phylogenetic tree showed that the IBDV variant clustered with the American and Chinese variant viruses and are highly comparable to the novel Chinese variants, with 99.9% identity. Based on the sequences and phylogenetic analyses, this is the first identification of an IBDV variant being reported in Malaysia. Further research is required to determine the pathogenicity of the IBDV variant and the protective efficacy of the current IBD vaccines being used against the virus.

## Introduction

Infectious bursal disease (IBD) is an acute poultry disease that affects young chickens mostly aged between 3 and 6 weeks. The condition has a global impact on the poultry industry, and various control measures are put in place to reduce losses. The causative agent, infectious bursal disease virus (IBDV) is a double-stranded non-envelope RNA virus that belongs to the family *Birnaviridae* (1). Two serotypes of IBDV have been recognized, and only serotype I viruses are pathogenic to chickens. Serotype I IBDV strains have varying antigenicity and pathogenicity, and are classified as classical, very virulent, and antigenic variants (2). Recently, the viruses have been classified into genogroups (3). The viral genome contains two segments; A and B (4). Segment A (~3.3 kb) has two partially overlapping open reading frames (ORF1 and ORF2), where the larger ORF1 encodes for precursor polyprotein (pVP2-VP4-VP3, 110 kDa), which is cleaved autoproteolytically by a serine protease, VP4, to yield the immature viral proteins pVP2 (48 kDa), VP3 (32 kDa), and VP4 (28 kDa). The immature pVP2 is subsequently cleaved at its C-terminus, thereby yielding the mature VP2 protein (40 kDa) and four peptides that remain linked in the viral particle (5). Whilst the smaller ORF2 encodes for a non-structural protein (VP5), segment B (2.8 kb) encodes for RNA-dependent RNA polymerase VP1. The polymerase is vital for encapsidation of the viral genome and RNA priming for synthesis (6). Both genome segments contribute to the pathogenicity of virulent viruses (7). Still, a previous report has shown that there was no increase in virulence when a reassortant strain was derived from classical (segment A) and very virulent (segment B) strains (8).

The VP2 protein is an essential gene of the virus as it carries the protective antigen (9, 10). It is the most widely studied gene, and has revealed the antigenic properties of IBDV (11–13). The VP2 protein is the major capsid protein of IBDV, containing major immunodominant epitopes, which are vital for inducing neutralizing antibodies against IBDV (9, 14). This conformational neutralizing domain is represented in HVR of the VP2 gene that spans from aa positions 206 to 350. The region has high mutation rates that determine virulence, tissue culture adaptation, and antigenic properties (10, 15). Also, it contains major hydrophilic peaks, A (212–224 aa) and B (312–324 aa), which flanked two hydrophobic aa residues, 248–254 and 279–290 (16). Variation in IBDV antigenicity mainly occurs in major peaks A and B (9). Even a single mutation at these loops can significantly affect the antigenicity of IBDV, rendering the available IBD vaccine ineffective (10, 17, 18).

Previous works on sequencing have identified amino acids on these structural loops that are characteristics of classical, variant, and very virulent IBDV. Classical strains have aa 222P, 249Q, 286T, and 318G, whilst 222A, 249Q, 286T, and 318G motifs characterize vvIBDV viruses (2, 10). In contrast, variant strains have specific aa residues 222T, 249K, 286I, and 318D (13, 16, 19).

Most of the research conducted on IBDV characterization has been done to establish the current status of the virus pathotype for better control strategies. Virus identification is usually achievable by sampling, detecting, and sequencing the immunodominant region of the virus. Several studies have reported the evolution of IBDV in some geographical locations around the world with the emergence of an antigenic variant (20, 21) and recombinant (22), reassortant (23, 24), and distinct (25, 26) strains of the virus. These isolated strains of IBDV with diverse antigenicity have made vaccination programmes against IBDV more complex due to vaccine failure. The presence of vvIBDV in Malaysia was first reported in 1991 when the partial VP2 gene was sequenced from a suspected IBD outbreak (27). Since then, the vvIBDV is often reported in commercial poultry farms despite vaccination. Most of these reported cases were based on a serological test (28), and the genetic information of these viruses is largely unknown. The serological test has the limitation of being a false positive, such that an escaped mutant could not be accounted for. Most of the molecularly characterized IBDV are limited to the VP2 gene (29–31), yet the complete genome has not been explored. The last complete IBDV genome sequences that were characterized were carried out between 2004 and 2008 (32).

Because of the high risk of emerging variant IBDV strains, the circulating field isolates should be characterized to update the genetic characteristics of the IBDV in recent years. We selected some isolates for full-length genome sequencing, which would provide more genetic information relevant to control strategies. Therefore, this study focuses on detecting and genome sequencing using the MiSeq platform of recent IBDV isolates from vaccinated flocks of broilers from 2017 to 2019. We hypothesized that mutations do exist amongst the current Malaysian IBDV strains.

## Materials and Methods

### Samples

Bursal samples were collected between 2017 and 2019 from 30 flocks of chickens suspected of IBD from various commercial broiler farms located in five states of Malaysia. The birds had a history of vaccination, and most of the IBD vaccines were administered when the bird was a day-old. The samples were collected when the bird was between 23 and 40 days old. The shortest time gap between vaccination and sample collection was 21 days. The bursal samples showed one or a combination of these lesions: congestion, hemorrhages, oedema, and atrophy. All the bursae were stored at −20°C until further analysis at the Laboratory of Vaccine and Biomolecules, Institute of Bioscience (IBS).

### Sample Preparation

The bursal samples were processed according to the method described by OIE (33). Briefly, a 20% bursal homogenate was prepared in phosphate-buffered saline (PBS, pH 7.4) using a homogeniser (Qiagen, Germany). The homogenate was followed by freeze-thawing three times, and it was centrifuged at 4,000 rpm (Eppendorf Centrifuge 5,810 R) at 4°C for 5 min. The supernatant was collected and filtered through a 0.45 μm filter (Sartorius, Germany) and stored in a −80°C freezer (Panasonic, Japan) until use.

### Virus Isolation

The samples that were stored at −80°C (Panasonic, Japan), were thawed and blind passaged two times in 10-day old specific pathogen-free (SPF) chicken eggs (single-comb White Leghorn) to obtain a pure culture for whole genome sequencing. The SPF embryonated chicken eggs (Thai SPF Co. Ltd.) were obtained from Malaysian Vaccine Pharmaceuticals (MVP). The chorioallantoic membrane (CAM) from the inoculated eggs of each virus isolate was separately harvested using sterile scissors and forceps in biosafety level 2.

### RNA Extraction

Total RNA was extracted from 250 μL of each CAM homogenate using TRIzol® reagent (Invitrogen, USA) following the manufacturer's instructions. Before the reverse transcription process of the IBDV RNA, the extracted dsRNA was denatured using dimethyl sulfoxide (DMSO), as described by OIE (33).

### Detection, RT-PCR, and Partial Segment Sequencing

The presence and identity of the IBDV in the bursal homogenates were confirmed with a RT-PCR protocol targeted at the hypervariable region (HVR) of the VP2 gene. Complementary DNA (cDNA) was generated from the denatured RNA template using the maxima first-strand cDNA synthesis kit (Thermo Scientific, Lithuania) according to the manufacturer's instructions. Previously described primers, forward 5′-GCCCAGAGTCTACACCAT-3′ and reverse 5′-CCCGGATTATGTCTTTGA-3′ (34), resulting in 743 bp flanking 737–1,479 nucleotides were used for detection, PCR amplification, and sequencing of the VP2 HVR of IBDV. The Phusion high-fidelity PCR master mix kit (Thermo Scientific, Lithuania) was used for the amplification following the manufacturer's instructions. The GeneJET Gel extraction kit (Thermo Scientific, Lithuania) was used for the DNA purification process. The purified PCR products were sequenced by the dideoxy-mediated chain-termination method, which was outsourced from Microgen Inc.

### RT-PCR of the Full-Length Genome of IBDV

Two sets of primers that amplify the nearly complete genome of segments A and B of the IBDV, were designed and optimized. The primers designated as F_A_-5′ GATACGATCGGTCTGACCCC-3′ and R_A_-5′ TGGGATGTTGTATGGCCGAA 3′ (A) and F_B_-5′ GACCCTCTGGGAGTCACGAA and R_B_-5′ AGGCGAAGGCCGGGGATA 3′ (B) amplified 3,235 bp and 2,800 bp of segments A and B, respectively. The Superscript IV™ one-step RT-PCR system kit (Invitrogen, USA) was used according to the manufacturer's instructions. Briefly, 50 μL volume reaction mix was prepared from 25 μL of 2x platinum superFi™ RT-PCR master mix, 10 μM Forward Primer 2.5 μL (0.5 μM), 10 μM Reverse Primer 2.5 μL (0.5 μM), RNA template 2.5 μL, superscript IV™ RT mix 0.5 μL, and top up 17 μL nuclease-free water. The mixture was briefly centrifuged and ran under PCR cycling conditions: one cycle at 50°C for 10 min, 98°C for 2 min, and 35 cycles at 98°C for 10 s, 64.5°C for 10 s, and 72°C for 1.40 min and another cycle at 72°C for 5 min (segment A). For segment B, one cycle at 50°C for 10 min, 98°C for 2 min, and 40 cycles at 98°C for 10 s, 66°C for 10 s, and 72°C for 1.30 min, and another cycle at 72°C for 5 min. The PCR products were extracted using the QIAquick Gel Extraction kit (QIAGEN, Germany) according to the manufacturer's protocol, and the DNA proceeded to ethanol precipitation.

### Full-Length Sequencing of Genomic Segments by Next-Generation Sequencing

Qubit™ dsDNA HS assay kit (Invitrogen, USA) was used for the accurate quantification of purified dsDNA of the PCR fragments derived from IBDV isolates. A total of 250–350 ng of the DNA fragments were subjected to library preparation using the Nextera flex DNA Sample Prep Kit (Illumina Inc., USA) according to the manufacturer's instructions. The DNA libraries were sequenced using the Illumina MiSeq platform (Illumina Inc., USA).

Reads were assessed, and a Phred quality score of 30 was used for the quality control employed in BBDuk software (BBTools v36). The excellent quality reads were then *de novo* assembled using MEGAHIT software (version 1.2.8) to obtain contigs. Aragon prodigal PROKKA software (version 1.14.0) was applied for the gene prediction function. Finally, the *de novo* assemblies and alignment on the reference sequences were compared.

### Phylogenetic Analyses

The HVR of the VP2 gene was used for the construction of a phylogenetic tree using a proposed new IBDV classification (3, 12). The evolutionary analyses were conducted using the maximum likelihood method and Kimura 2-parameter model (35), and the tree was constructed in MEGA-X (36) software following muscle alignment, and 1,000 bootstraps were applied. The analysis involved 54 nucleotide sequences with a total position of 423 in the final dataset, including the reference strains of IBDV detected in different countries (Supplementary Table 1). The TempEst v1.5.3 software was used to estimate the mutation rate in the HVR dataset. The sequences cover the IBDV isolates collected from 1967 to 2019.

The complete genome sequences of the five isolates and reference strains (Supplementary Table 2) from GenBank were used for the construction of a phylogenetic tree. The evolutionary analyses were conducted using the maximum likelihood method and the General Time Reversible model (GTR) (37) for segment A. Tamura-Nei model (TN93) model (38) was applied for segment B. The trees were constructed in MEGA-X (36) software following muscle alignment and 1,000 bootstraps. The analysis involved 25 nucleotide sequences with a total position of 3,260 and 2,781 nts for segment A and segment B, respectively, in the final dataset, including the reference strains.

### Estimation of Selection Pressure

The selection pressures on the complete coding sequences of VP5, polyprotein (PP), and VP1 proteins were estimated using Tajima's D neutrality test in MEGA X. The selection pressures of the overall and site specific selection was also determined by a non-synonymous (dN) to a synonymous (dS) nucleotide substitution rate per codon using the single-likelihood ancestor counting (SLAC) and fixed-effects likelihood (FEL) methods from an online Datamonkey website (http://www.datamonkey.org). Values of dN/dS <1, =1, and >1 indicate a purifying selection, neutral selection, and diversifying selection, respectively.

## Results

### RT-PCR and Sequencing of IBDV Hypervariable Region

**Figure 1:**
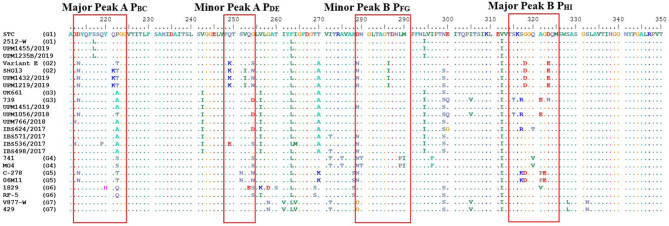
Deduced amino acid sequences of the VP2 hypervariable region from aa position 211 to 350 of IBDV strains. Dots indicate aa positions identical to UPM1455/2019. Major and minor hydrophilic peaks are boxed with a red line.

Out of the 30 bursal samples investigated, positive RT-PCR results were obtained from 11 samples. The RT-PCR confirmed the presence of IBDV in the inoculated embryos, and the 743-bp-long nucleotide and 247 amino acids sequence, flanking the HVR, were characterized for the 11 IBDV isolates. However, only the 420 bp nucleotide and 140 aa sequence of the VP2 HVR of the IBDV isolates were used for the sequence analyses (Figure 1). All the detected and sequenced strains of IBDV covered for the nucleotides positions 625–1,044 and amino acids positioned 211–350 according to the numbering system of Bayliss et al. (14) of the HVR that characterized the isolates.

The nucleotide identity matrix of UPM1455/2019 and UPM1235B/2019 was 97.9–99.7% identical to the IBDVs of genogroup 1 (G1) (STC and 2512-W). The identity of UPM1432/2019 and UPM1219/2019 isolates was 94.6–98.3% identical, compared to genogroup 2 (G2) (Variant E and SHG13). The other seven isolates of the present study shared identity with the vvIBDVs which belongs to genogroup 3 (G3) (UK661 and 739).

### Amino Acids Sequence and Phylogenetic Analyses for HVR

The deduced amino acid sequences are shown in Figure 1. Based on the partial amino acid sequences of VP2 HVR, two strains matched the strains in the G1, two isolates aligned with the isolates of G2, and seven isolates were similar to the strains in the G3 (Figure 1).

The two isolates; UPM1455/2019 and UPM1235B/2019, shared 100% amino acid identity with each other. These isolates demonstrated the amino acids sequence typical of classical strains, STC, and 2512-W (Figure 1). Other residues were: 212D, 213D, 217L, 242V, 256V, 270T, and 299N of the HVR. The amino acid residues are similar to that of STC and 2512-W strains except for the substitution L217S in the STC strain. Therefore, the isolates have a higher amino acid identity to STC and 2512-W strains.

However, isolates UPM1432/2019 and UPM1219/2019 had amino acid substitutions suggestive of G2 (Figure 1). Some characteristic amino acids were also similar to American and Chinese variant strains, Variant E and SHG13, including 213N, 242V, 253Q, 279N, 284A, 294L, 323E, and 330S, found in these two isolates (Figure 1).

Meanwhile, characteristic amino acids typical of very virulent IBDV strains were found in the remaining seven isolates, except for isolate UPM1056/2018, which had an A222T change (Figure 1). In addition, the isolate showed some atypical amino acid substitutions, which includes D213N, G254D, S315T, S317R, and A321E which have been reported in some Malaysian vvIBDV strains, 739, 866, UPM08PF3, and UPM08PF4. The IBS624/2017 strain also contained similar amino acid substitutions at G254D and S317R. However, the isolate had two unique mutations—E300G and Q320T (Figure 1). Specifically, the UPM766/2018 strain was identical to the global vvIBDV strains with only one amino acid change, D212N, which is commonly found in some Malaysian vvIBDV strains (UPM08MF1 and UPM04/190). The isolates IBS498/2017, IBS5362017, IBS571/2017, and UPM1451/2019 had a D279N change which has been detected in vvIBDV strains of Jordan (710) and Nigeria (IBDV80). IBS498/2017 and IBS571/2017 isolates had an I272T mutation. Q219P, Q249E, and G254S substitutions were observed in IBS536/2017 isolate, where Q219P is unique (Figure 1). The Q249E change was found in some Malaysian vvIBDV strains, UPM04/190, UPM04178, and UPM04238, while G254S was detected in some global vvIBDV strains (IBDV/NIE/95/001c, 100,056, SA-KZN95, UPM08MF1, and UPM94273) and strains of variant subtypes.

The VP2 HVR of all the study isolates maintains the serine-rich heptapeptide sequences, SWSASGS, the virulence marker, which is adjacent to the major hydrophilic peak B, P_HI_ (Figure 1, red box).

**Figure 2:**
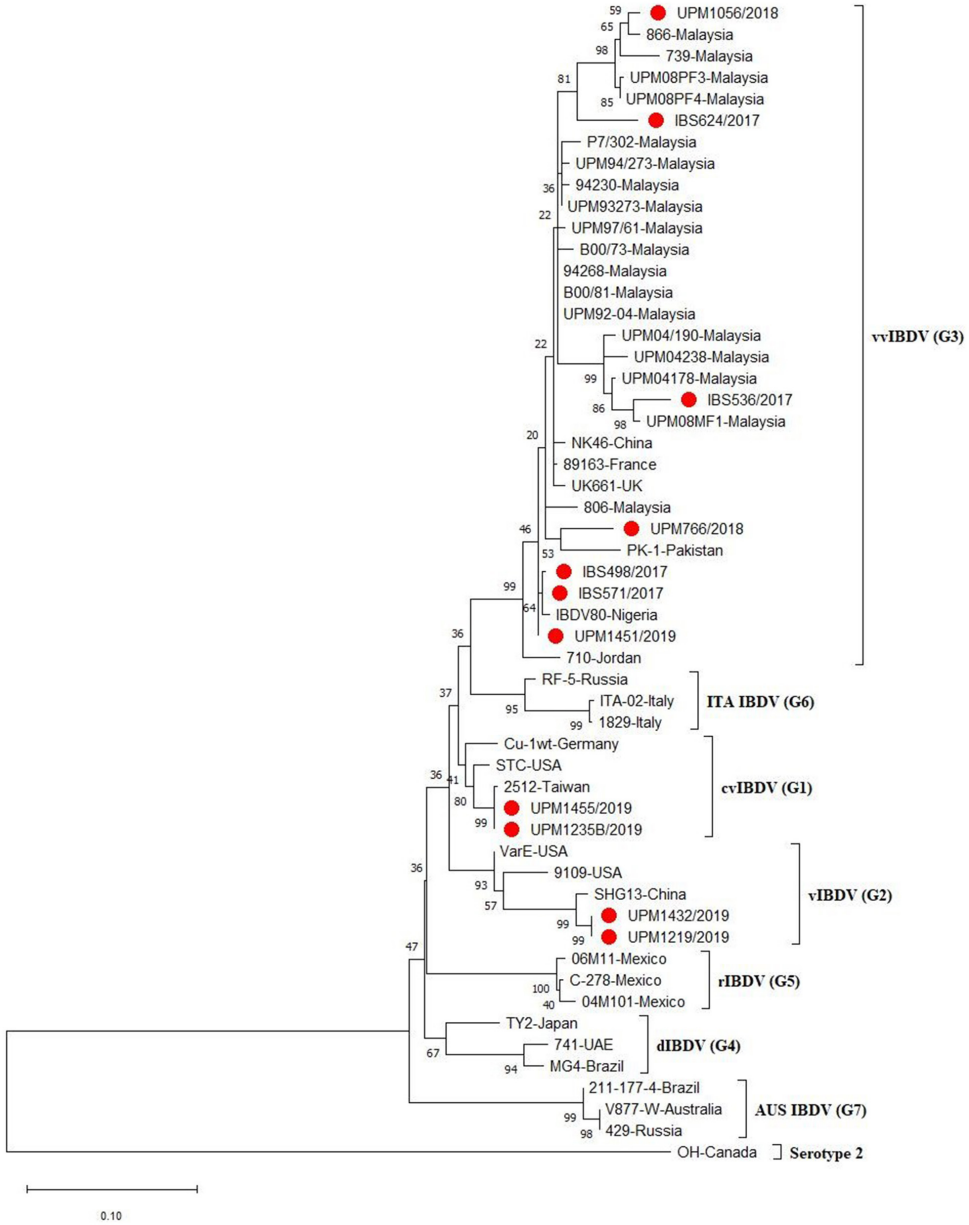
Maximum Likelihood-based Phylogenetic tree of the hypervariable region of IBDV using 433 nucleotides fragment with the Kimura 2-parameter model. Each genogroup and the serotype 2 strains are indicated with different colors. Bootstrap = 1,000 replicates. The filled circles indicate strains identified in this study.

A phylogenetic tree constructed from the aligned sequences of HVR showed that the sequences formed seven major clusters, which corresponds to the seven proposed genogroups (3). Seven (IBS498/2017, IBS571/2017, IBS536/2017, IBS624/2017, UPM766/2018, UPM1056/2018, UPM1451/2019) out of the 11 sequences formed a cluster with the G3, vvIBDV, which are commonly found globally (Figure 2, bootstrap value 99%). It is worth noting that the vvIBDV strain clustered with the vvIBDV strain of Europe (UK661, 89,163), Asia (HK46, PK-1), Africa (IBDV80), and the Middle East (710). The IBS624/2017 and UPM1056/2018 isolates formed sub-clusters with the more recent Malaysian vvIBDV strain (UPM08PF3, UPM08PF4, 739, 866) (Figure 2, bootstrap value 81%). Two sequences (UPM1235B/2019 and UPM1455/2019) formed a cluster with G1, cvIBDV (STC, Cu-1wt and 2,512). In contrast, the remaining two sequences (UPM1219/2019 and UPM1432/2019) formed a distinct cluster with vaIBDV (G2) (Variant E, 9109 and SHG13) (Figure 2, bootstrap value 93%). The estimated mutation rate was found to be 8.463 × 10^−4^ per year. The HVR sequences of the isolates have been submitted to GenBank with accession numbers MT431209-MT431219.

### Complete Genome Analysis

#### Nucleotide Sequence Analysis

The RT-PCR reactions, using the designed primers, successfully amplified the complete genome of the five IBDV isolates. The nearly full-length consensus sequences of both segments A and B of IBS536/2017, IBS624/2017, UPM766/2018, UPM1056/2018, and UPM1432/2019 were determined by NGS. The sequence results of segment A of IBS536/2017, IBS624/2017, UPM766/2018, UPM1056/2018, and UPM1432/2019 isolates contained 3,219, 3,195, 3,200, 3,201, and 3,235 bp, respectively, which encompasses two overlapping ORFs (3085 bp) except for UPM766/2018 which has 3,073 bp. The coding and non-coding regions are shown in Table 1. The sequenced segment B of all the isolates consists of 2,799 bp of RNA dependent RNA polymerase (VP1) with coding ORF (2,640 bp), flanked by 5′-UTR (83 bp) and 3′-UTR (56 bp) (Table 2). The genome sequences of the isolates have been deposited in GenBank with accession numbers MT505339 to MT505343 (segment A) and MT505344 to MT505348 (segment B).

**Table 1:** Complete genome annotation sequences of segment A of the study IBD viruses.

**Isolates**	**Type**	**Accession number**	**5^**′**^ UTR (nts)**	**VP5 (nts)**	**VP2-4-3 (nts)**	**3^**′**^ UTR (nts)**
IBS536/2017	vv	MT505339	1–73	74–538	120–3,158	3,159–3,219
IBS624/2017	vv	MT505340	1–43	44–493	90–3,128	3,129–3,195
UPM766/2018	vv	MT505341	1–60	61–498	95–3,133	3,134–3,200
UPM1056/2018	vv	MT505342	1–49	50–499	96–3,134	3,135–3,201
UPM1432/2019	va	MT505343	1–83	84–533	130–3,168	3,169–3,235

**Table 2:** Complete genome annotation sequences of segment B of the study IBD viruses.

**Isolates**	**Type**	**Accession number**	**5^**′**^ UTR (nts)**	**VP1 (nts)**	**3^**′**^ UTR (nts)**
IBS536/2017	vv	MT505344	1–83	84–2,723	2,724–2,779
IBS624/2017	vv	MT505345	1–83	84–2,723	2,724–2,779
UPM766/2018	vv	MT505346	1–83	84–2,723	2,724–2,779
UPM1056/2018	vv	MT505347	1–83	84–2,723	2,724–2,779
UPM1432/2019	va	MT505348	1–83	84–2,723	2,724–2,779

The segment A nucleotide coding sequence of the variant strain, UPM1432/2019, was 95.4 and 98.2% identical to variant E and SHG19, respectively. UPM1056/2018, UPM766/2018, and IBS624/2017 sequences were 95–96% identical to the Malaysian vvIBDV strain (UPM08MF1, UPM04/190, UPM97/61, UPM94/273) and the European vvIBDV strain, UK661. The IBS536/2017 strain was 96–98% identical to the Asian and European vvIBDV strains.

The segment B nucleotide coding sequence of the variant strain, UPM1432/2019, was 95.1 and 96.4% identical to variant E and SHG19, respectively. vvIBDV strains, UPM1056/2018, UPM766/2018, IBS624/2017, and IBS536/2017 have 96–97% nucleotides identity to UPM08MF1, UPM04/190, UPM97/61, UPM94/273, and UK661 strains.

#### Amino Acid Sequences Analysis

The VP5 protein of the study viruses contained 149 amino acids as seen in other vvIBDV strains (UPM97/61, UPM94/273, UK661, and HK46). However, isolate UPM766/2018 showed only 145 amino acids, missing the first four residues (MLSL) that are commonly present in most vvIBDV strains. A similar mutation was obtained in HuN11, IBD13HeB01, and SK53 strains. However, the IBS536/2017 strain has 154 amino acids with an additional five residues, RTDRC, at its C-terminal which are similar to those found in UPM08MF1 and UPM04/190 strains.

The amino acid sequence of the VP5 gene of the variant strain, UPM1432/2019, was 89.9 and 95.3% identical to Variant E and SHG19, respectively. The VP5 amino acid sequence of the vvIBDV strain UPM1056/2018 was 94.1% identical to UPM08MF1 and UPM04/190 strains and 97.3% identical to the UPM97/61 and UPM94/273 strain, while it was 97.9% identical to the UK661 strain. The UPM766/2018 strain had 90.2% amino acid identity to UPM08MF1 and UPM04/190 strains and 93.2–94.6% identity to UPM97/61, UPM94/273, and UK661 strains. Strain IBS624/2017 showed 92.8% identity to UPM08MF1 and UPM04/190 strains and 97.3% identity to UPM97/61 and UPM94/273 strains, whilst it had 96.6% identity to the UK661 strain. The IBS536/2017 strain VP5 gene was 100% identical to UPM08MF1 and UPM04/190 strains and 94.1–96.1% identical to UPM97/61, UPM94/273, and UK661 strains.

The VP5 gene of variant strain UPM1432/2019, had three unique amino acid mutations—P102L, G109C, and W131R. Additionally, substitutions at S53P and T135A were found in the Chinese variant (SHG358 strain). The amino acid substitutions S7R, S44P, Q92R, C104G, and K147E observed in the UPM1432/2019 strain, were obtained in the SHG19 strain. In addition, E124K was detected in variant E and SHG19 strains (Table 3). A few unique aa changes were observed in some positions of the VP5 gene of the study vvIBDV strains. These amino acids are T135A in the UPM1056/2018 strain, N12D, and T135I in the UPM766/2018 strain. The mutations V69I and H127Y were found in the IBS624/2017 isolate. Another amino acid substitution at position 78L of the UPM1056/2018 and IBS536/2017 isolates was detected in UPM08MF1, UPM04/190, UK661, and SHG19 (Table 3).

**Table 3:** The characteristic amino acids in the VP5 gene of IBDV.

**Strain**	**VP5**															
	**7**	**12**	**44**	**53**	**69**	**78**	**92**	**99**	**102**	**104**	**109**	**124**	**127**	**131**	**135**	**147**
Variant-E	S	N	S	S	V	I	Q	S	P	C	G	K	H	W	T	K
UPM1432/2019	R	N	P	P	*	L	R	*	L	G	C	*	*	R	A	E
SHG19	R	*	P	*	*	L	R	P	*	G	R	*	*	*	*	E
UK661	*	*	*	*	*	L	*	*	*	*	*	E	*	*	*	*
UPM1056/2018	*	*	*	*	*	L	*	*	*	*	*	E	*	*	A	*
UPM766/2018	*	D	*	*	*	F	*	*	*	*	*	E	*	*	I	*
IBS624/2017	*	*	*	*	I	F	*	*	*	*	*	E	Y	*	*	*
IBS536/2017	*	*	*	*	*	L	*	*	*	*	*	E	*	*	*	*
UPM08MF1	*	*	*	*	*	L	*	*	*	*	*	E	*	*	*	*
UPM04/190	*	*	*	*	*	L	*	*	*	*	*	E	*	*	*	*
UPM97/61	*	*	*	*	*	F	*	*	*	*	*	E	*	*	*	*
UPM94/273	*	*	*	*	*	F	*	*	*	*	*	E	*	*	*	*
OKYM	*	*	*	*	*	F	*	*	*	*	*	E	*	*	*	*
HK46	*	*	*	*	*	F	*	*	*	*	*	E	*	*	*	*
F52/70	G	*	*	*	*	*	*	*	*	*	*	E	*	*	*	*
Cu-1 wt	*	*	*	*	*	*	*	*	*	*	*	E	*	*	*	*
CEF94	*	*	*	*	*	*	*	*	*	*	*	E	*	*	*	*

*The gray area with asterisk indicate aa position identical to the reference strain, Variant E*.

The polyprotein (PP) of the study IBDV strains consists of the pVP2 protein (512 amino acids), the VP4 protein (243 amino acids), and the VP3 protein (257 amino acids). The amino acid sequence of the PP for the variant strain, UPM1432/2019, was 97.3 and 99.9% identical to Variant E and SHG19, respectively. The amino acid sequence of the UPM1056/2018 strain was similar to the previously characterized Malaysian and other vvIBDV strains (UPM08MF1, UPM04/190, UPM97/61, UPM94/273, and UK661). However, the UPM766/2018 and IBS624/2017 strains had 98.4–98.9% amino acid identity to the Malaysian vvIBDV strains. The IBS536/2017 strain had 99% amino acid identity to the UPM08MF1 and UPM04/190 strains.

Analysis of the VP2 protein of the selected viruses confirmed the VP2 HVR sequences obtained using the Sanger's method. Although, the VP2 protein contains the HVR, it has amino acid mutations at positions other than the HVR. The VP2 sequence of the variant strain, UPM1432/2019, revealed amino acid substitutions at T73I, N77D, and I187V, which are present in the SHG19 strain. The N79S amino acid change was found in the UPM04/190 and SHG19 strains (Table 4). The VP2 gene for the vvIBDV, UPM1056/2018 strain showed amino acid substitutions at T73I, which is commonly detected in the Chinese variant IBDV. Another substitution at position T359K was found in the UPM1056/2018 strain, and the amino acid was reported in IR01, a vvIBDV strain from Iran. Strain IBS624/2017 had V384I substitution. The IBS536/2017 strain also presented one mutation at N79S, which is similar to the UPM04/190 and SHG19 strains (Table 4).

**Table 4:** Amino acid substitutions in the polyprotein of the study IBDV (excluding HVR) in comparison to reference strains.

**Strain**	**VP2**							**VP4**												**VP3**								
	**73**	**77**	**79**	**187**	**359**	**384**	**451**	**532**	**544**	**553**	**596**	**642**	**680**	**685**	**686**	**715**	**734**	**751**	**754**	**815**	**819**	**890**	**905**	**919**	**982**	**987**	**990**	**1,008**
Variant-E	T	N	N	I	T	V	I	V	G	R	R	K	C	K	I	P	L	H	M	R	A	Y	L	E	P	K	A	D
UPM1432/2019	I	D	S	V	*	*	L	*	*	*	*	*	*	*	*	*	*	*	*	K	*	*	*	*	*	*	*	*
SHG19	I	D	S	V	*	*	L	*	*	*	*	*	*	*	*	*	*	*	*	*	*	*	*	*	*	*	*	*
UK661	*	*	*	*	*	*	L	*	*	*	*	*	Y	N	V	S	*	D	*	*	*	*	*	G	*	*	V	*
UPM1056/2018	I	*	*	*	K	*	L	I	*	K	*	N	Y	S	V	S	*	D	*	*	S	*	P	D	*	R	*	E
UPM766/2018	*	*	*	*	*	I	L	*	*	*	*	*	Y	N	V	S	F	D	*	*	*	F	*	*	*	R	*	*
IBS624/2017	*	*	*	*	*	I	L	*	S	K	*	N	Y	N	V	S	*	D	*	*	*	*	*	*	S	*	V	*
IBS536/2017	*	*	S	*	*	*	L	*	*	S	K	*	Y	N	V	S	*	D	L	*	*	*	*	*	*	*	V	*
UPM08MF1	*	*	*	*	*	*	L	*	*	*	*	*	Y	N	V	S	*	D	*	*	*	*	*	*	*	*	*	E
UPM04/190	*	*	S	*	*	*	L	*	*	*	*	*	Y	N	V	S	*	D	*	*	*	*	*	*	*	*	V	*
UPM97/61	*	*	*	*	*	*	L	*	*	*	*	N	Y	N	V	S	*	D	*	*	*	*	*	*	*	*	V	*
UPM94/273	*	*	*	*	*	*	L	*	*	*	*	N	Y	N	V	S	*	D	*	*	*	*	*	*	*	*	V	*
OKYM	*	*	*	*	*	*	L	*	*	*	*	*	Y	N	V	S	*	D	*	*	*	*	*	*	*	*	V	*
HK46	*	*	*	*	*	*	L	*	*	*	*	*	Y	N	V	S	*	D	*	*	*	*	*	*	*	*	V	*
F52/70	*	*	*	*	*	*	*	*	*	*	*	R	*	*	V	*	*	*	*	*	*	*	*	*	*	*	*	*
Cu-1 wt	*	*	*	*	*	*	*	*	*	*	*	*	*	*	V	*	*	*	*	*	*	*	*	*	*	*	*	*
CEF94	*	*	*	*	*	*	*	*	*	*	*	*	*	*	V	*	*	*	*	*	*	*	*	*	*	*	*	*

*The gray area with asterisk indicate aa position identical to the reference strain, Variant E*.

The VP4 protein (513-755 amino acids) of the study isolates revealed some amino acid changes. Analysis of the VP4 protein of the variant isolate, UPM1432/2019, showed five amino acid substitutions. Four changes, Y680C, N685K, S715P, and D751H were detected in the IBDV variant (Variant-E and SHG19) and classical IBDV (F52/70, Cu-1 wt, and CEF94), while V686I substitution was only present in Variant-E, SHG19 (Table 4). Some unusual amino acid mutations, V532I, R553K, and N685S, were observed for the UPM1056/2018 isolate. The VP4 protein of strain UPM766/2018 is typical of vvIBDV, except for the amino acid at position L734F—which is unique. Isolate IBS624/2017 had one unique and one uncommon amino acid substitution, G544S and R553K, respectively. Moreover, we found three unique amino acid mutations at R553S, R596K, and M754L in the IBS536/2017 strain (Table 4).

The VP3 protein (756-1012 amino acids) of the isolates presented some exceptional amino acid substitutions. The variant strain, UPM1432/2019, had one specific amino acid change at R815K, and the changes at L922Q and I951L were reported in SHG19. The UPM1056/2018 strain had four unique amino acid substitutions, L781M, A819S, L905P, E919D, and K987R (Table 4). Although, the isolate UPM766/2018 VP3 amino acid sequence was highly comparable to most reported vvIBDV, it exhibited unique mutations at Y890F and K987R. The VP3 amino acid sequence of the IBS624/2017 strain had two rare substitutions, P982S, and P996S. The IBS536/2017 isolate also had one unusual change, I951V, which is also found in UK661 (Table 4).

Segment B of the study isolates contained 879 amino acids for RNA dependent RNA polymerase (VP1). Various amino acid substitutions were identified in the VP1 protein.

The amino acid sequence of the polymerase for the variant strain, UPM1432/2019, was highly identical to the SHG19 strain. The amino acid sequence of vvIBDV, UPM1056/2018, was 99.3% identical to UPM08MF1, 99.2% (UPM04/190 and UPM97/61), 98.6% to UPM94/273, and 98.7% identical to UK661 strains. However, the UPM766/2018, IBS624/2017, and IBS536/2017 strains had 98.1–98.8% amino acid identities to the UPM08MF1, UPM04/190, UPM97/61, UPM94/273, and UK661 strains.

Analysis of the VP1 amino acid sequence of variant UPM1432/2019, revealed two unique substitutions, K21R and E688D, and the amino acid mutation A24V was found in the HK46 and SHG19 strains. The C595S and T859I strains are also common to the Uruguayan and Argentian dIBDV. The alterations V141I, 147D, and D240E, are common to the SHG19 strain. Similarly, the isolate had A163V, which is found in the UPM97/61 and SHG19 strains, while E515D was reported in Variant E and SHG19 (Table 5). The vvIBDV isolates, UPM766/2018, IBS624/2017, and IBS536/2017, had rare substitutions at amino acid positions K13T, A83S, K508R, L553M, and C595S. In addition, UPM1056/2018, IBS624/2017, and IBS536/2017 had one uncommon amino acid substitution at A664T (Table 5). However, the five study isolates had a common amino acid at 4I, which is commonly found in variant, classical, and some Malaysian vvIBDV strains (Table 5). The characteristic vvIBDV signature of TDN was also found in the four isolates, while NED characterized the variant strain.

**Table 5:** Amino acid substitutions in the VP1 protein of the study IBDV in comparison to reference strains.

**Strain**	**VP1**																											
	**4**	**13**	**21**	**24**	**61**	**83**	**141**	**145**	**146**	**147**	**163**	**240**	**242**	**287**	**390**	**393**	**508**	**511**	**515**	**553**	**562**	**595**	**646**	**664**	**687**	**688**	**695**	**859**
Variant-E	I	K	K	A	V	A	V	N	E	G	A	D	D	T	L	E	R	R	D	L	S	C	G	A	S	E	K	T
UPM1432/2019	*	*	R	V	*	*	I	*	*	D	V	E	*	*	*	*	*	*	*	*	*	S	*	*	*	D	*	I
SHG19	*	*	*	V	*	*	I	*	*	D	V	E	*	*	*	*	*	*	*	*	*	*	*	*	*	*	*	*
UK661	V	*	*	*	I	*	*	T	D	N	*	*	E	A	M	D	*	S	E	*	P	*	S	*	P	*	R	*
UPM1056/2018	*	*	*	*	I	*	*	T	D	N	*	*	E	A	M	D	*	S	E	*	P	*	S	T	P	*	R	*
UPM766/2018	*	T	*	*	I	S	*	T	D	N	*	*	E	A	M	D	R	S	E	M	P	S	S	*	P	*	R	*
IBS624/2017	*	T	*	*	I	S	*	T	D	N	*	*	E	A	M	D	R	S	E	M	P	S	S	T	P	*	R	*
IBS536/2017	*	T	*	*	I	S	*	T	D	N	*	*	E	A	M	D	R	S	E	M	P	S	S	T	P	*	R	*
UPM08MF1	*	*	*	*	I	*	*	T	D	N	*	*	E	A	M	D	*	S	E	*	P	*	S	*	P	*	R	*
UPM04/190	*	*	*	*	I	*	*	T	D	N	*	G	E	A	M	D	*	S	E	*	P	*	S	*	P	*	R	*
UPM97/61	V	*	*	*	I	*	*	T	D	N	P	*	E	A	M	D	*	S	E	*	P	*	S	*	P	*	R	*
UPM94/273	V	*	*	*	I	*	*	T	D	N	*	*	E	A	M	D	*	S	E	*	P	*	S	*	P	*	R	*
OKYM	V	*	*	*	I	*	*	T	D	N	*	*	E	A	M	V	*	S	E	*	P	*	S	*	P	*	R	*
HK46	V	*	*	V	I	*	*	T	D	N	*	*	E	A	M	D	*	S	E	*	P	*	S	*	P	*	R	*
F52/70	*	*	*	*	*	*	*	*	*	*	*	*	*	*	*	*	R	*	E	*	*	*	*	*	*	*	*	*
Cu-1 wt	*	*	*	*	*	*	*	*	*	*	*	*	*	*	*	*	R	*	E	*	*	*	*	*	*	*	*	*
CEF94	*	T	*	*	*	*	*	*	*	*	*	*	*	*	*	*	R	*	E	*	*	*	*	*	*	*	*	*

*The gray area with asterisk indicate aa position identical to the reference strain, Variant E*.

### Genome-Based Phylogenetic Tree

**Figure 3:**
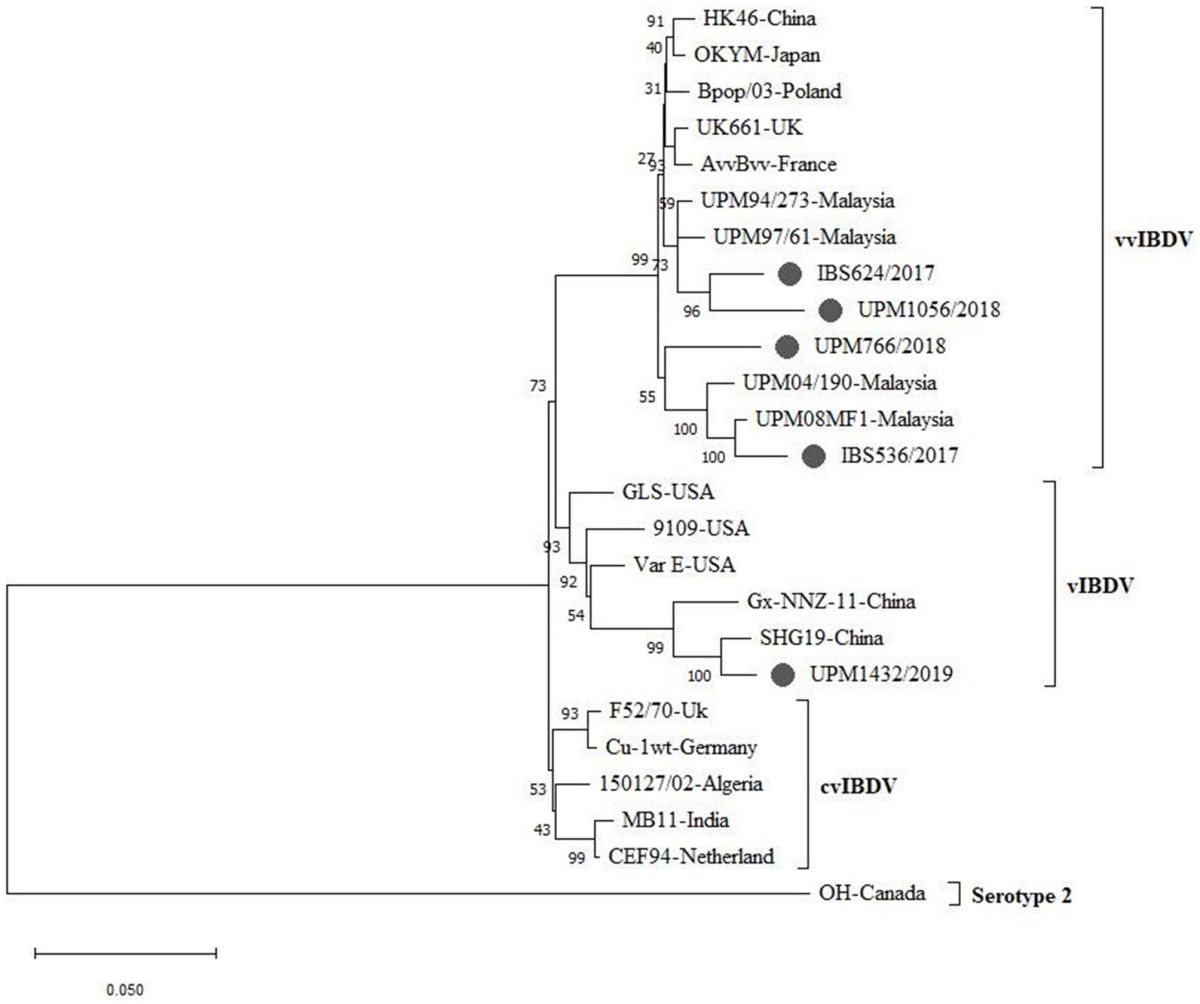
Maximum Likelihood-based Phylogenetic tree of complete segment A genome of IBDV using 3,260 nucleotides fragment with the General Time Reversible model with Gamma distribution. Each cluster is indicated with a different color. Bootstrap = 1,000 replicates. The filled circles indicate strains identified in this study.

A phylogenetic tree was constructed from the aligned nucleotide sequences of the complete segment A of the study strains and the reference IBDV strains retrieved from GenBank. The tree showed that the sequences formed three major clusters distinct from the outgroups. Four vvIBDV strains (IBS536/2017, IBS624/2017, UPM766/2018, UPM1056/2018) out of the five study sequences formed a cluster with the vvIBDV found globally (Figure 3, bootstrap value 99%). It is worth noting that the vvIBDV clustered with the vvIBDV of Europe (UK661, AvvBvv, Bpop/03) and Asia (HK46, OKYM, UPM08PF1, UPM04/190, UPM97/61, and UPM94/273). The IBS536/2017 and UPM766/2018 strains formed a clade with the recently characterized Malaysian vvIBDV strains (UPM08PF1 and UPM04/190), while IBS624/2017 and UPM1056/2018 strains formed sub-clusters with the earlier characterized Malaysian vvIBDV strains (UPM97/61 and UPM94/273) (Figure 3, bootstrap value 99%). In contrast, UPM1432/2019 formed a distinct cluster with the American and Chinese variant IBDV (GLS, Variant E, 9,109, Gx-NNZ-11, and SHG19) (Figure 3, bootstrap value 93%).

**Figure 4:**
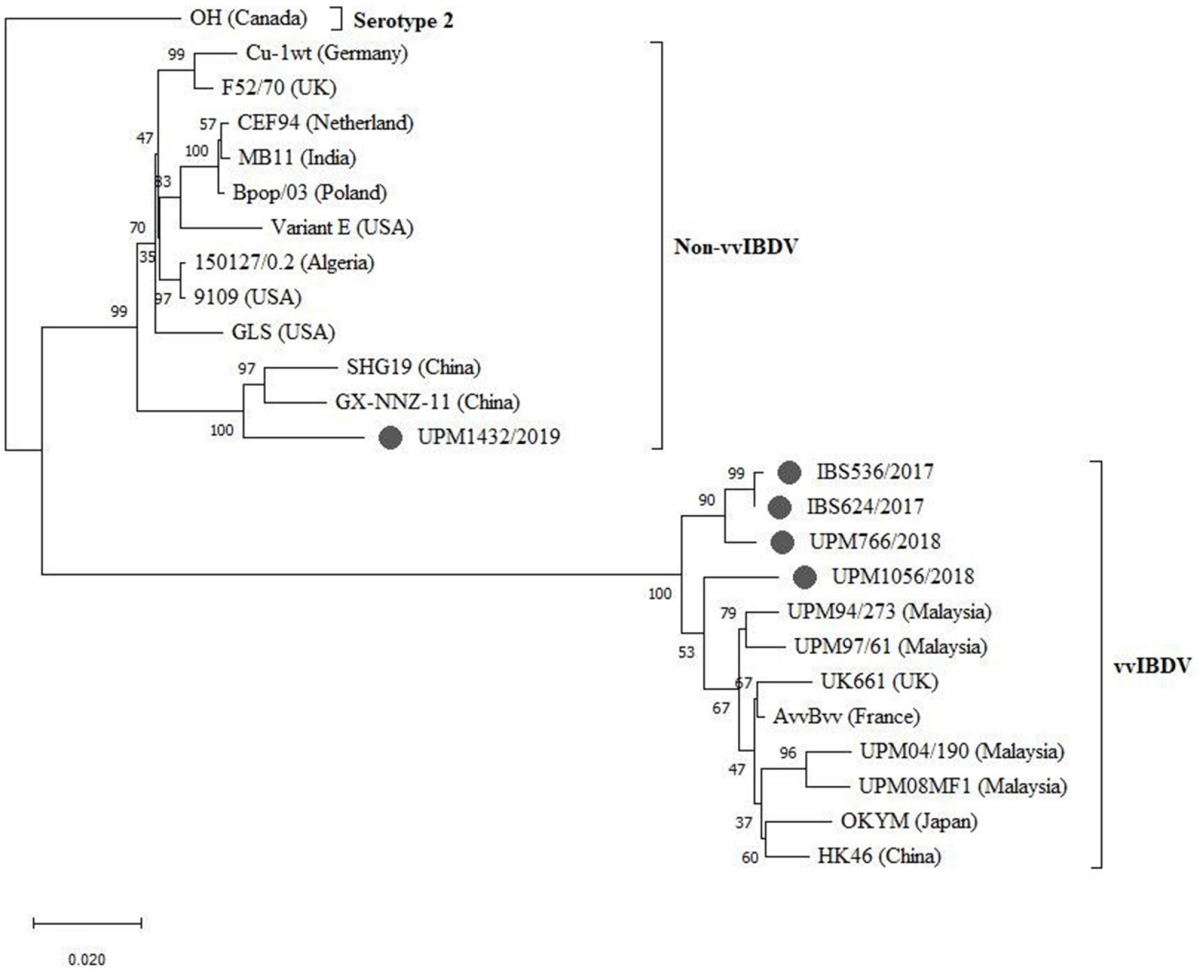
Maximum Likelihood-based Phylogenetic tree of complete segment B genome of IBDV using 2,781 nucleotides fragment with the Tamura-Nei model with Gamma distribution. Each cluster is indicated with a different color. Bootstrap = 1,000 replicates. The filled circles indicate strains identified in this study.

The sequenced segments B of the isolates, IBS536/2017, IBS624/2017, UPM766/2018, and UPM1056/2018, formed a cluster with segment B of vvIBDV found in various regions (Figure 4, bootstrap value 100%). Although, three of the isolates formed sub-clusters, they are more related to the European and Asian strains (UK661, AvvBvv, HK46, OKYM, UPM08PF1, UPM04/190, UPM97/61, and UPM94/273).

The UPM1056/2018 strain formed a distant relationship with the previously reported Malaysian and other vvIBDV strains (Figure 4). However, strain UPM1432/2019 formed a distinct cluster with the non-vvIBDV groups, including classical and antigenic variant viruses. Interestingly, the isolate formed a distinct sub-cluster with the Chinese novel variant IBDV (Gx-NNZ-11 and SHG19) (Figure 4, bootstrap value 100%).

### Selection Pressure Analysis

The neutrality test for most of the genes showed a negative D value with no significance (*P* > 0.1) (Table 6). The SLAC and FEL analyses demonstrate that most of the sites deviated significantly (*P* < 0.1) from neutrality to a negative selection. However, one site (13) of HVR, three sites (78,116, and 135) of VP5, two sites (222 and 990) of PP, and one site (24) of the VP1 gene presented positive selection (*P* < 0.1). Almost all the positive selections were shown by the less conservative FEL method (Table 6). The distribution of codon under selection showed the lowest negative selection in VP5 (3.2%), and the highest negative selection was found in PP (36.8%). The greater percentage of substitutions across all the genes were on a random selection with no constraint (neutral).

**Table 6:** Codon selection pressures on IBDV sequences used in the study.

**Protein Sequences**	**Tajima's D**	**dN/dS**	**Positively selected sites**		**Negatively selected sites**	
			**SLAC (codon)**	**FEL (codon)**	**SLAC**	**FEL**
HVR	−0.6453	0.133	1 (13)	1 (13)	52	76
VP5	−1.562	1.710	0	3 (78, 116, 135)	2	5
PP	−1.055	0.822	0	2 (222, 990)	187	372
VP1	0.788	0.059	0	1 (24)	125	295

*Positively and negatively selected sites are estimated at 90% confidence level*.

## Discussion

We performed molecular detection and characterization of IBDVs, isolated between 2017 to 2019, from the bursa of Fabricius of vaccinated commercial flocks of broilers in different parts of Malaysia. The isolated IBDV strains were from broiler flocks with a history of vaccination against IBD, low feed conversion ratio, suspected clinical signs of IBD, and with a mortality of <8%, while some samples showed no obvious clinical manifestations. Economic losses occurred through immunosuppression induced by the viruses. Some reports have shown that variant viruses replicate in the presence of immunity to classical strains and cause severe bursal damage (39). Several molecular techniques have been applied by various laboratories to characterize the antigenic variant of IBDV (19, 40, 41). This antigenic drift has been attributed to eight aa changes at the HVR of the capsid protein (19).

The nucleotides and aa sequences of the VP2 HVR of each of the 11 studied Malaysian isolates revealed the highest similarity to the respective classical, variant, and vvIBDV strains. Comparatively, the nucleotide sequence analysis of the study vvIBDV isolates and the existing Malaysian vvBDV strains present a varying identity, which suggests the genetic diversity of the virus in circulation, as antigenic variations have occurred in more recent IBDV viruses. Genetic diversity is not uncommon, considering that RNA viruses undergo high mutation rates due to poor proofreading activity of the RNA polymerase (42).

The deduced amino acid sequences of the study isolates have revealed the characteristics of each isolate. Notably, the VP2 remains the antigenic determinant of IBDV; therefore, it is widely used for the molecular characterization of the virus (43, 44). In the HVR, the eight amino acid substitutions (213N, 222T, 249K, 254S, 270A, 286I, 318D, and 323E) described for the variant E strain (19) were identical to UPM1219/2019 and UPM1432/2019 strains except for one amino acid substitution, S254N, which was reported in a Chinese novel variant, SHG13 strain (20). Even though the amino acid 249K and 254S were regarded as unique substitutions for the American variants (18), not all variants contain both amino acids (45). Jackwood et al. (13) considered aa 222T and 254S as the hallmark for variant E/Del. Interestingly, the P_HI_ loop aa 318D and 323E have also been commonly reported in the American variant (46). In addition to the eight amino acid substitutions for the variant, two more observed substitutions, Q221K and V252I, at the P_BC_ and P_DE_ loop structure, respectively, were also found in the Chinese variant strain. These mutations may affect the antigenicity of the strain since they occurred in the antigenic sites, which induces neutralizing Ab to the viruses (10).

In contrast, the amino acid residues observed for the isolates UPM1235B/2019 and UPM1455/2019 were typical of classical strains. For that, the strains could probably have genetic and antigenic characteristics to classical strains (vaccine strain). Although, the strains were similar to the vaccine (immune complex, 2512-W) used in the flock, the samples were collected 40 days post vaccination. However, a report has shown that the vaccine virus could be detected 15 days post vaccination (47). On the other hand, key amino acid positions in seven isolates, which includes 222A, 242I, 256I, 284A, 294I, and 299S, are typical of vvIBDV strains (48), suggesting that the seven isolates belong to genogroup 3. The amino acid at position 222 is an essential residue because of its location at the tip of the P_BC_ loop. An amino acid change at position 222 could result in vaccine failure (49). Contrary to most vvIBDV, the UPM1056/2018 strain possesses D213N and A222T substitutions which are conserved in variant and recombinant IBDV strains. The finding of A222T substitutions is not uncommon in genogroup 3 viruses as some vvIBDV have been reported to have such mutations while conserving other residues for very virulent strains (3). Some previous reports demonstrated an A222S mutation in vvIBDV from Asia, a YV strain (50) and Tasik strain (51), yet they retained very virulent properties. The amino acid change D279N observed in vvIBDV, IBS498/2017, IBS536/2017, IBS571/2017, and UPM1451/2019 strains were similar to what was reported in Chinese vvIBDV strains, DMS and NC (50). The finding, in combination with A284T substitution, has been shown to influence cell culture tissue adaptation of vvIBDV (50). The report of Lim et al. (52) demonstrated the adaptation of a vvIBDV (HK46 strain) to chicken embryonic fibroblast by site-directed mutagenesis of D279N and A284T residues. Another mutagenesis study showed that mutations at Q253H and A284T contributed significantly to vvIBDV tissue adaptation (53). However, a more recent work reported that a mutation at position D279N could facilitate vvIBDV adaptability to chicken embryonated eggs and BGM-70 cultures (54).

On the other hand, 315T, 317R, and 321E are unique mutations to the VP2 gene of UPM1056/2018 and some recent Malaysian vvIBDV (3), except that 321E was previously reported in Columbian and Venezuelan IBDV (55) and some American IBDV variant. The molecular implication of these mutations is unknown, therefore, a mutagenesis study could verify their functions. Interestingly, the mutations occur at the major hydrophilic peak, P_HI_, which is known to induce neutralizing antibodies. The most crucial aa mutations for the IBS624/2017 isolate were E300G and S317R, which are unique. Like strain UPM1056/2018, the 317R occurred in the P_HI_ loop, which may generate non-neutralizing Ab recognition (10). Although, two out of the three amino acid changes (219P, 249E, and 254S) observed for the IBS536/2017 strain were previously reported in a few vvIBDV. Some amino acid mutations observed for the study IBDV strains that occurred within the VP2 gene appeared unique and significantly contributed to the genetic diversity of the Malaysian IBDV.

Furthermore, we compared the nearly complete nt and deduced amino acid sequences of IBS536/2017, IBS624/2017, UPM766/2018, UPM1056/2018, and UPM1432/2019 strains with previously reported whole-genome sequences of IBDV strains. Even though the percentage of genome coverage was 98–99.2%, the genetic characteristics of the IBDV strains have been identified. Only some parts of the non-coding sequences from both segments were missing for the isolates. Genetic diversity among Malaysian IBDV was evident when the most recent isolated strains showed varying nt and similar amino acid sequence identities to the previous Malaysian IBDV strains.

Amino acid substitutions that are observed in the VP5 gene of the variant, UPM1432/2019 and the other vvIBDV strains, may affect the protein function. A previous study reported that the VP5 gene contributes to the pathogenicity of IBDV-infected cells through late apoptotic processes (56). According to previous work, a mutant IBDV with a deleted VP5 gene showed significant bursal lesion reduction compared to other wild IBDV (57). Site-directed mutagenesis can prove the functional mechanism of the few amino acid changes observed in UPM1056/2018, UPM766/2018, IBS624/2017, and IBS536/2017 strains. Interest may be given to leucine substitution that is observed at position 78 of UPM1432/2019, UPM1056/2018, and IBS536/2017 where the report showed the possibility of function alteration when isoleucine substituted phenylalanine (58).

The PP of the UPM1432/2019 strain is 99.9% identical to the Chinese variant (SHG19); therefore, they may share similar genetic and antigenic properties. The characterization of SHG19 as being a novel IBDV variant was only recently performed (20), and the strain was mostly reported with additional amino acid substitutions that were not discovered in the American variant (variant E) (9). For PP sequences of the vvIBDV strains, UPM1056/2018, UPM766/2018, IBS624/2017, and IBS536/2017, <99% amino acid sequence identity in comparison to Malaysian vvIBDV could indicate the evolutionary relationship among the Malaysian vvIBDV.

The earlier discussions on the HVR of the 11 study isolates were informative; however, the VP2 genes of the complete genomes appeared to have amino acid substitutions aside from the variable region. The VP2 remains the essential gene that significantly determines the genetic and antigenic properties of the virus (9, 10, 44). The VP2 gene of the UPM1432/2019 strain contains amino acid substitutions that are characteristic of variant strains (9, 20, 55). Like the SHG19 strain, the UPM1432/2019 strain may share similar antigenic characteristics (39). Four amino acid substitutions Q221K, V252I, S254N, and N299S, contributed to the difference between the Malaysian variant strains and the American variant E.

The protease, VP4 protein, plays a critical role in the catalytic activity of PP that uses a Ser^652^/Lys^692^ catalytic dyad in the active sites (59). Interestingly, the catalytic dyad is conserved within all the study viruses. Some mutations, which occurred within the catalytic triad of the UPM1432/2019 strain, are conserved in previously reported variant strains (39). Even though mutations R553K and N685S, that are recognized with a vvIBDV UPM1056/2018 strain, are detected in some vvIBDV, 553R is required for the suppression of IFN-β expression and glucocorticoid-induced leucine zipper (GILZ) protein ubiquitylation (60). Based on the available literature, amino acid substitutions unique for IBS624/2017 (544S) and IBS536/2017 (553S, 596K and 754L) have not been reported, and their molecular functions remain unclear. However, amino acid mutation M754L for IBS536/2017 is located at the protease cleavage site, VP4/VP3 (^754^MAA^756^); as a result, it may affect the proteolytic activity of the serine protease. The single unique amino acid change, L734F, in VP4 of the UPM766/2018 strain has also not been reported, however, the impact of the difference can be determined through site-directed mutagenesis.

In the VP3 domain (756–852 amino acids), the unique amino acid substitutions found in UPM1432/2019 (R815K) and UPM1056/2018 (L781M and A819S) may affect the homomeric interaction of the VP3 protein (61). In our study, isolate UPM1056/2018 showed an amino acid substitution D1008E located in the RdRp-binding domain (62), where VP3 binds to VP1 for virus replication and assembly. The amino acid 1005A for the UPM1432/2019 strain is also a characteristic of vvIBDV (32) but mutation A1005T has been reported for the variant E strain. In the proposed dsRNA-binding domain (976–1,002 amino acid), we found 990A in UPM1056/2018 and UPM766/2018 vvIBDV strains, a characteristic residue of variant and classical strains (63). The finding of the one unique amino acid change, 987R, for both UPM1056/2018 and UPM766/2018 vvIBDV located in the dsRNA-binding domain has not previously been reported, therefore, its molecular function can be validated by site-directed mutagenesis.

The VP1 protein contributes significantly to the virulence and pathogenicity of IBDV (7). Most of the mutations that occurred within the VP1 protein for the UPM1432/2019 strain were not involved with the active site of the central polymerase; consequently, they may have less implication to the VP1 viral genome. Even though, the amino acid substitutions happened to be conserved in a variant and classical strains, the function of the unique arginine substitution at position 21 is yet to be understood. The previous finding demonstrated that the VP1 N-terminal possesses the putative guanylylation site residue 166S, which is implicated in protein priming (64).

Similarly, the rare amino acid substitution, C595S, that was found in the variant strain and three other vvIBDV, is located at the thumb of the central polymerase. The residue is mostly detected in distinct IBDV from Uruguay and Argentina (65). Of the four isolates with the characteristic TDN tripeptides at positions 145/146/147, respectively, UPM1056/2018, IBS624/2017, and IBS536/2017 strains presented mutation at position A664T. This substitution falls within the 27-residue C-terminal of the polymerase, and the motif is said to implicate conformation of the 3D structure of the polymerase active site (64). Some observed uncommon amino acid changes might have little or no effect on the characteristics of the vvIBDV strains, therefore, the possible role of these mutations on virulence should be determined through site-directed mutagenesis. A critical finding in segment B of all the study Malaysian IBDV is the V4I aa substitution, which was reported to reduce viral replication in SPF and attenuates the pathogenicity of the virus (66).

Phylogenetic analysis revealed that the study and the reference viruses formed seven distinct clusters according to the seven new genogroup classifications of IBDV (3). Based on the nucleotide sequences of HVR, the tree showed that the Malaysian study IBDV isolates grouped with mostly recognized global IBD viruses. The two strains (UPM1235B/2019 and UPM1455/2019) formed a cluster to G1. It further confirmed that the two identified IBDVs belong to G1 of classical viruses, which could be derived from vaccine strains currently in use in the Malaysian poultry industry.

However, two other strains that formed a cluster with the American and Chinese variant strains could be considered as the emerging Malaysian variant IBDV. Therefore, most of the IBDVs identified in this study grouped with the G3, which includes not only the vvIBDV but their reassortant pathotypes (3).

The phylogenetic tree of segment A sequences indicates that the IBS536/2017 is closely related to the Malaysian vvIBDV, UPM04/190, and UPM08FM1 that were isolated in 2004 and 2008, respectively, while the UPM766/2018 isolate, isolated in 2018, was distantly related to the two reference Malaysian strains. The IBS624/2017 and UPM1056/2018 strains indicated more relatedness to the UPM94/273 and UPM97/61 strains isolated in 1994 and 1997, respectively. Interestingly, the IBS624/2017 and UPM1056/2018 strains showed a close relationship to European and other Asian vvIBDV strains. Therefore, the phylogeny suggests that the four strains are vvIBDVs. The interest could be seen in UPM1056/2018, which showed a more recent evolution time among these viruses. Nevertheless, the UPM1432/2019 strain in the phylogeny formed a distinct cluster with the Chinese and American variant IBDV; therefore, it could be considered a variant strain.

Compared with the typical vvIBDV, the recent Malaysian strains, IBS536/2017, IBS624/2017, and UPM766/2018, showed differences in segment B where they grouped in a distant branch from all other reference vvIBDVs. Also, the UPM1056/2018 isolate indicates a distant relationship to the other three vvIBDVs in the current study and vvIBDVs in the reference group. The findings suggest that the current IBDV with a very virulent signature may have additional mutations that placed them in a distant monophyletic clade. Furthermore, the UPM1432/2019 isolate position in the phylogenetic tree characterized the strain with a non-vvIBDV signature. Although, the strain clustered with the non-vvIBDV, it is more closely related to the Chinese variant (20).

Interestingly, 1/3 of the total codons of PP and VP1 appeared to be evolving under purifying selection. The negatively selected sites were supported by the dN/dS values of 0.082 and 0.059, respectively. The phenomenon might be an indication that the PP and VP1 were subjected to restrictions, and are therefore relatively stable to high sequence variations. Although, the amino acids involved in virulence determinants are prone to variation, they are not associated with virion subunit interactions (67). However, the diversifying selection that was found in PP happened to be in the VP2 and VP3 capsid proteins. Codon 222 A to P, P to T, P to S, P to Q of VP2 is located in the hydrophilic P_BC_ loop, a critical region for antigenic variation within IBDVs (10). The site is also under a constant selection by the host immunity (68). Codon 990 (A to V and V to A) of VP3 is located in the dsRNA binding domain. The region is not quite unique to particular strains, as classical, variant, and vvIBDV have been reported with either of the amino acid residues (69). Similarly, a more diverse selection occurs in VP5 that is not associated with virulence or antigenicity, but a report has shown that a mutant virus with deleted VP5 showed reduced virulence (57). Virus evolution is better understood by the estimation of substitution rates. Genetic diversity restriction within a population of viruses results in lower adaptability and pathogenicity. The estimated rate of 8.46 × 10^−4^ per year of HVR is in line with the finding of Gao et al. (70), who reported a mutation rate of 0.9 × 10^−4^ substitution per year of VP2 gene for IBDVs. The mutation rate observed in the HVR of IBDV is not unexpected, as various substitutions are evolving due to continuous vaccine selection pressure, thereby allowing adaption to the viral environment.

## Conclusions

In conclusion, the present study describes the complete sequences of some recent Malaysian IBDV isolates and demonstrates that most of the circulating viruses are of a very virulent strain with an antigenic drift. Based on the sequences and phylogenetic analyses, the first identification of an IBDV variant in Malaysia is reported. The study also provides genetic information that could be useful for control of the disease.

## Data Availability Statement

The data presented in the study are deposited in the (National Centre for Biotechnology Information) repository, accession number [MT431209 to MT431219, MT505339 to MT505343 (segment A) and MT505344 to MT505348 (segment B)].

## Author Contributions

HA, AI, MH-B, and AO conceived the research. infectious bursal disease virus isolation, and propagation, RNA extraction, RT-PCR, sample library preparation, and data analysis were conducted by HA and supervised by AI, AO, and MH-B. The manuscript was written by HA, AI, AO, and MH-B. The paper was finalized and submitted by HA. All authors reviewed and approved the submitted manuscript.

## Conflict of Interest

The authors declare that the research was conducted in the absence of any commercial or financial relationships that could be construed as a potential conflict of interest.
