# Supplementary material for: Genetic Diversity of Recent Infectious Bursal Disease Viruses Isolated From Vaccinated Poultry Flocks in Malaysia
Source: Front Vet Sci. 2021 Apr 20;8:643976. doi: 10.3389/fvets.2021.643976 (PMC8093787; doi:10.3389/fvets.2021.643976)
Supplement: Supplementary file 1 [file Data_Sheet_1.docx]

**Supplementary Table 1: Reference sequences used for the construction of a phylogenetic tree using hypervariable region**

| **S/N** | **Strain/Isolate (Collection date)** | **Accession number** | **Country** | **Genogroups** | **Classification** |
| --- | --- | --- | --- | --- | --- |
| 1 | STC (1967) | D00499 | USA | 1 | Classical |
| 2 | 2512 (2018) | MH329181 | USA | 1 | Classical |
| 3 | Cu-1 wt (1975) | AF362747 | Germany | 1 | Classical |
| 4 | Var E (1994) | AF133904 | USA | 2 | Variant |
| 5 | 9109 (2003) | AY462027 | USA | 2 | Variant |
| 6 | SHG13 (2017) | MH879090 | China | 2 | Variant |
| 7 | UPM92-04 (1992) | AF262030 | Malaysia | 3 | Very virulent |
| 8 | UPM93273 (1993) | AY245550 | Malaysia | 3 | Very virulent |
| 9 | 94230 (1994) | AY520911 | Malaysia | 3 | Very virulent |
| 10 | 94268 (1994) | AY333088 | Malaysia | 3 | Very virulent |
| 11 | UPM94/273 (1994) | AF248612 | Malaysia | 3 | Very virulent |
| 12 | UPM97/61 (1997) | AF247006 | Malaysia | 3 | Very virulent |
| 13 | P97/302 (1997) | AF464901 | Malaysia | 3 | Very virulent |
| 14 | B00/73 (2000) | AY520909 | Malaysia | 3 | Very virulent |
| 15 | B00/81 (2000) | AY520910 | Malaysia | 3 | Very virulent |
| 16 | UPM04178 (2004) | AY970665 | Malaysia | 3 | Very virulent |
| 17 | UPM04190 (2004) | AY791998 | Malaysia | 3 | Very virulent |
| 18 | UPM04238 (2004) | DQ000436 | Malaysia | 3 | Very virulent |
| 19 | UPM08MF1 (2007) | GQ131544 | Malaysia | 3 | Very virulent |
| 20 | UPM08PF3 (2007) | GQ131540 | Malaysia | 3 | Very virulent |
| 21 | UPM08PF4 (2007) | GQ131541 | Malaysia | 3 | Very virulent |
| 22 | 739 (2016) | MF142568 | Malaysia | 3 | Very virulent |
| 23 | 806 (2016) | MF142584 | Malaysia | 3 | Very virulent |
| 24 | 866 (2017) | MF142587 | Malaysia | 3 | Very virulent |
| 25 | UK661 (1989) | NC_004178 | UK | 3 | Very virulent |
| 26 | HK46 (1998) | AF092943 | China | 3 | Very virulent |
| 27 | 89163 (1989) | HG974563 | France | 3 | Very virulent |
| 28 | PK-1 (2014) | KT281984 | Pakistan | 3 | Very virulent |
| 29 | IBDV80 (2011) | JX424079 | Nigeria | 3 | Very virulent |
| 30 | 710 (2015) | MF142560 | Jordan | 3 | Very virulent |
| 31 | TY2 (2002) | LC136880 | Japan | 4 | distinct |
| 32 | 741 (2016) | MF142569 | UAE | 4 | distinct |
| 33 | MG4 (2009) | JN982252 | Brazil | 4 | distinct |
| 34 | 06M11 (2006) | JQ277695 | Mexico | 5 | Recombinant |
| 35 | C-278 (1999) | AF498627 | Mexico | 5 | Recombinant |
| 36 | 04M101 (2004) | DQ916210 | Mexico | 5 | Recombinant |
| 37 | RF-5 (1994) | Z97002 | Russia | 6 | ITA |
| 38 | ITA-02 (2011) | JN852986 | Italy | 6 | ITA |
| 39 | 1829 (2011) | KY930929 | Italy | 6 | ITA |
| 40 | 211-177-4 (2011) | KY612971 | Brazil | 7 | Australian |
| 41 | V877-W (2009) | HM071991 | Australia | 7 | Australian |
| 42 | 429 (2015) | MF142536 | Russia | 7 | Australian |
| 43 | OH (1982) | U30818 | USA | - | Serotype 2 |

**Supplementary Table 2: Reference IBDV strains used for the phylogenetic tree construction using complete genome**

| **S/N** | **Strain** | **Accession # Segment A** | **Accession # Segment B** | **Country** | **Genogroup** |
| --- | --- | --- | --- | --- | --- |
| 1 | F52/70 | HG974565 | HG974566 | UK | 1 |
| 2 | Cu-1 wt | AF362747 | AF362748 | Germany | 1 |
| 3 | CEF94 | AF194428 | AF194429 | Netherland | 1 |
| 4 | MB11 | KU891986 | KU891987 | India | 1 |
| 5 | 150127/02 | MF969107 | MF969108 | Algeria | 1 |
| 6 | Variant E | AF133904 | AF133905 | USA | 2 |
| 7 | 9109 | AY462027 | AY459321 | USA | 2 |
| 8 | GLS | AY368653 | AY368654 | USA | 2 |
| 9 | SHG19 | MN393076 | MN393077 | China | 2 |
| 10 | GX-NNZ-11 | JX134483 | JX134484 | China | 2 |
| 11 | UPM08MF1 | KU516686 | KU516687 | Malaysia | 3 |
| 12 | UPM04/190 | KU958716 | KU958717 | Malaysia | 3 |
| 13 | UPM97/61 | AF247006 | AF527040 | Malaysia | 3 |
| 14 | UPM94/273 | AF527039 | AF527038 | Malaysia | 3 |
| 15 | UK661 | NC_004178 | NC_004179 | UK | 3 |
| 16 | OKYM | D49706 | D49707 | Japan | 3 |
| 17 | HK46 | AF092943 | AF092944 | China | 3 |
| 18 | Bpop/03 | MH545934 | MH545935 | Poland | 3 |
| 19 | AvvBvv | MG489892 | MG489893 | France | 3 |
| 20 | OH | U30818 | U20950 | USA | Serotype 2 |
